# Relationship between running performance and weather in elite marathoners competing in the New York City Marathon

**DOI:** 10.1038/s41598-022-25901-z

**Published:** 2022-12-08

**Authors:** Katja Weiss, David Valero, Elias Villiger, Mabliny Thuany, Ivan Cuk, Volker Scheer, Beat Knechtle

**Affiliations:** 1 Institute of Primary Care, University of Zurich, Zurich, Switzerland; 2 Ultra Sports Science Foundation, Pierre-Benite, France; 3 Klinik für Allgemeine Innere Medizin, Kantonsspital St. Gallen, St. Gallen, Switzerland; 4 Centre of Research, Education, Innovation and Intervention in Sport (CIFI2D), Faculty of Sport, University of Porto, Porto, Portugal; 5 Faculty of Sport and Physical Education, University of Belgrade, Belgrade, Serbia; 6 Medbase St. Gallen am Vadianplatz, Vadianstrasse 26, 9001 St. Gallen, Switzerland

**Keywords:** Environmental impact, Occupational health

## Abstract

It is well known that weather and pacing have an influence on elite marathon performance. However, there is limited knowledge about the effect of weather on running speed in elite marathoners. The aim of the present cross-sectional study was to investigate potential associations between running speed and weather variables in elite runners competing in the ‘New York City Marathon’ between 1999 and 2019. Data from all official female and male finishers with name, sex, age, calendar year, split times at 5 km, 10 km, 15 km, 20 km, 25 km, 30 km, 35 km, 40 km and finish and hourly values for temperature (°Celsius), barometric pressure (hPa), humidity (%) and sunshine duration (min) between 09:00 a.m. and 04:00 p.m. were obtained from official websites. A total of 560,731 marathon runners' records were available for analysis (342,799 men and 217,932 women). Pearson and Spearman correlation analyses were performed between the average running speed and the weather variables (temperature, pressure, humidity and sunshine). Ordinary Least Squares (OLS) regressions were also performed. The runner´s records were classified into four performance groups (all runners, top 100, top 10 and top 3) for comparison. Differences in running speed between the four performance groups were statistically significant (p < 0.05) for both men and women. Pearson (linear) correlation indicated a weak and positive association with humidity in the top 10 (r = 0.16) and top 3 (r = 0.13) performance groups that the running speed of the elite runners was positively correlated with humidity. Regarding sunshine duration, there was a weak and positive correlation with the running speed of the elite groups (r = 0.16 in the top 10 and r = 0.2 in the top 3). Spearman correlation (non-linear) identified a weak but negative correlation coefficient with temperature in all runners’ groups. Also, non-linear positive correlation coefficients with humidity and sunshine can be observed in the Spearman matrixes. A Multivariate Ordinary Least Squares (OLS) regression analysis showed no predictive power of weather factors. For elite runners competing in the ‘New York City Marathon’ between 1999 and 2019, the main findings were that elite runners became faster with increasing humidity and sunshine duration while overall runners became slower with increasing temperature, increasing humidity and sunshine duration. Weather factors affected running speed and results but did not provide a significant predictive influence on performance.

## Introduction

The ‘New York City Marathon’ has been held since 1970^1^ and is nowadays the largest city marathon in the world^2^. The race became of scientific interest during the last decade focusing on overuse injuries^3–5^. Another topic was physiological aspects (e.g., critical velocity) as predictors for marathon running performance^6^.

Since the number of master athletes increased in the ‘New York City Marathon’ in the last decades^7–11^, other research domains became of interest, such as pacing by age group marathoners^12–15^, pacing of elite runners^16^, age of peak marathon performance^17–19^ and sex differences in marathon running performance^20^.

A relatively new topic for the ‘New York City Marathon’ was the investigation of the influence of weather on marathon running performance^21,22^. It has been shown that faster runners were more significantly impacted by higher temperatures than slower runners under the same thermal conditions^22^. A study investigating 1,280,557 finishers in the ‘New York City Marathon’ competing between 1970 and 2019 showed that temperature on race day was positively associated, while wind speed and humidity were negatively associated with race time. Of all considered weather variables, temperature showed the most significant impact on race times. Temperature, humidity and wind speed had different influences on performance regarding the age and sex of the runners^21^.

Although we have a good insight into the pacing strategies of marathoners competing at different performance levels in the ‘New York City Marathon’^12–16^, there is no knowledge of whether the weather influences the running speed of these runners, especially the elite runners. Therefore, the aim of this study was to investigate the potential relationship between running speed and weather variables in elite runners competing in the ‘New York City Marathon’. Based upon existing knowledge^22^, we hypothesized that elite marathoners would be influenced by higher temperatures, i.e., higher temperatures would negatively influence running speed in faster runners.

## Methods

### Ethical approval

The institutional review board of Ethikkommission Kanton St. Gallen, Switzerland, approved this study (EKSG 01/06/2010). Since the study involved the analysis of publicly available data, the requirement for informed consent was waived.

### Strengthening the reporting of observational studies in epidemiology

The authors followed the STROBE checklist.

### The race

The ‘New York City Marathon’ is the largest city marathon in the world. The race takes place annually on the first Sunday of November in New York City. The course is a point-to-point course, starting at Fort Wadsworth on Staten Island, going through Brooklyn, Queens and the Bronx and then finishing at Central Park in Manhattan. The start of the race is in waves due to the large number of participants. The first start is at 08:00 a.m. with the professional wheelchair division, and at 08:22 a.m. is the start of the handcycle category and selected athletes with disabilities. Then, at 08:40 a.m., the start of the professional women, and at 09:05 a.m., the start of the professional men is held. From 09:10 a.m. to 12:00, five waves with recreational athletes are held. The race was not held in 2012 due to Hurricane Sandy and 2021 due to the COVID-19 pandemic.

### Weather data

Historical weather data in hourly intervals were obtained from Weather History Download New York^23^. The weather values available are hourly readings (between 09:00 a.m. and 04:00 p.m.) of the following magnitudes: temperature (°Celsius; measured at 2 m above ground), barometric pressure (hPa), humidity (%; measured at 2 m above ground) and sunshine duration (min). Between 1999 and 2019, no rain has been ever recorded during the ‘New York City Marathon’.

### Subjects

Data were obtained from the race website^24^ and included name, sex, age, calendar year, and split times at 5 km, 10 km, 15 km, 20 km, 25 km, 30 km, 35 km, and 40 km for all finishers of both sexes. Non-finishers, wheelchairs and handcycles were excluded from the analysis. A total of 560,731 marathon runners’ records were available for analysis (342,799 men (61.2%) and 217,932 women (38.8%)). The race times are recorded by a computer chip attached to the back of the runner's number, which calculates the difference between the race start and the point of reference^24^.

### Data processing

The processing of the data files involved several steps. First, the data was cleaned up and its integrity was verified by removing formatting errors and ensuring the alignment of the processed values. Next, each qualifying record had the time-adjusted average values of the weather factors imputed. Since the duration of each runner's race is different, from just over 2 h for elite runners to 6 or 8 h and over for the slowest participants, the average values of the temperature, pressure, and other weather factors they experienced during the race are also slightly different. Considering these differences when calculating and imputing the weather values to each record, we were able to better represent the actual environmental conditions in each case. Finally, the runner´s records were classified into four performance groups for comparison: all runners, top 100, top 10 and top 3. The top 3 sub-population is created by extracting the three best (fastest) athletes (both sexes) from each year's race. Similarly, the top 10 is created by extracting the best (fastest) ten male and ten female finishers from each year's race. The top 100 is created by extracting the best (fastest) 100 male and 100 female finishers from each year's race. These groups are not exclusive, so one record in the top 3 will also be included in the subsequent groups (top 10, top 100 and all runners). Because of this, the groups are nested where the top 100 includes the top 10, including the top 3. After the processing was complete, descriptive statistical methods were used to compare the groups and draw insights and conclusions.

### Statistical analysis

Descriptive statistics were presented in mean (standard deviation), minimum, maximum and percentiles (25, 50 and 75). Pearson and Spearman correlation was performed to analyze the average running speed and the weather variables (i.e., temperature, pressure, humidity and sunshine). The effect sizes of the correlations were 0–0.1 = no effect, 0.1–0.3 = small effect, 0.3–0.5 = medium effect and 0.5–1 = large effect, following Cohen^25^. Also, the distributions of running speeds for each performance group were calculated and displayed with boxplots for easy comparison. Similarly, with the range of weather conditions for each group, the Kolmogorov–Smirnov two-sample test was used to assess the statistical significance of the differences observed, given the unbalanced nature of the nested performance groups. Ordinary Least Squares (OLS) regression was performed to build a predictive model of the average race speed as a function of the weather factors. All analyses were done using the Python programming language (Python Software Foundation, https://www.python.org/) in a Google Colab notebook (https://colab.research.google.com/).

## Results

A total of 560,731 marathon runners’ records were available for analysis [342,799 men (61.2%) and 217,932 women (38.8%)]. As for the sampled down groups, the top 100 included 1300 male and 1300 female runners’ records, the top 10 included 130 male and 130 female runners’ records, and the top 3 groups included 39 male and 39 female runners’ records.

### Weather

**Figure 1:**
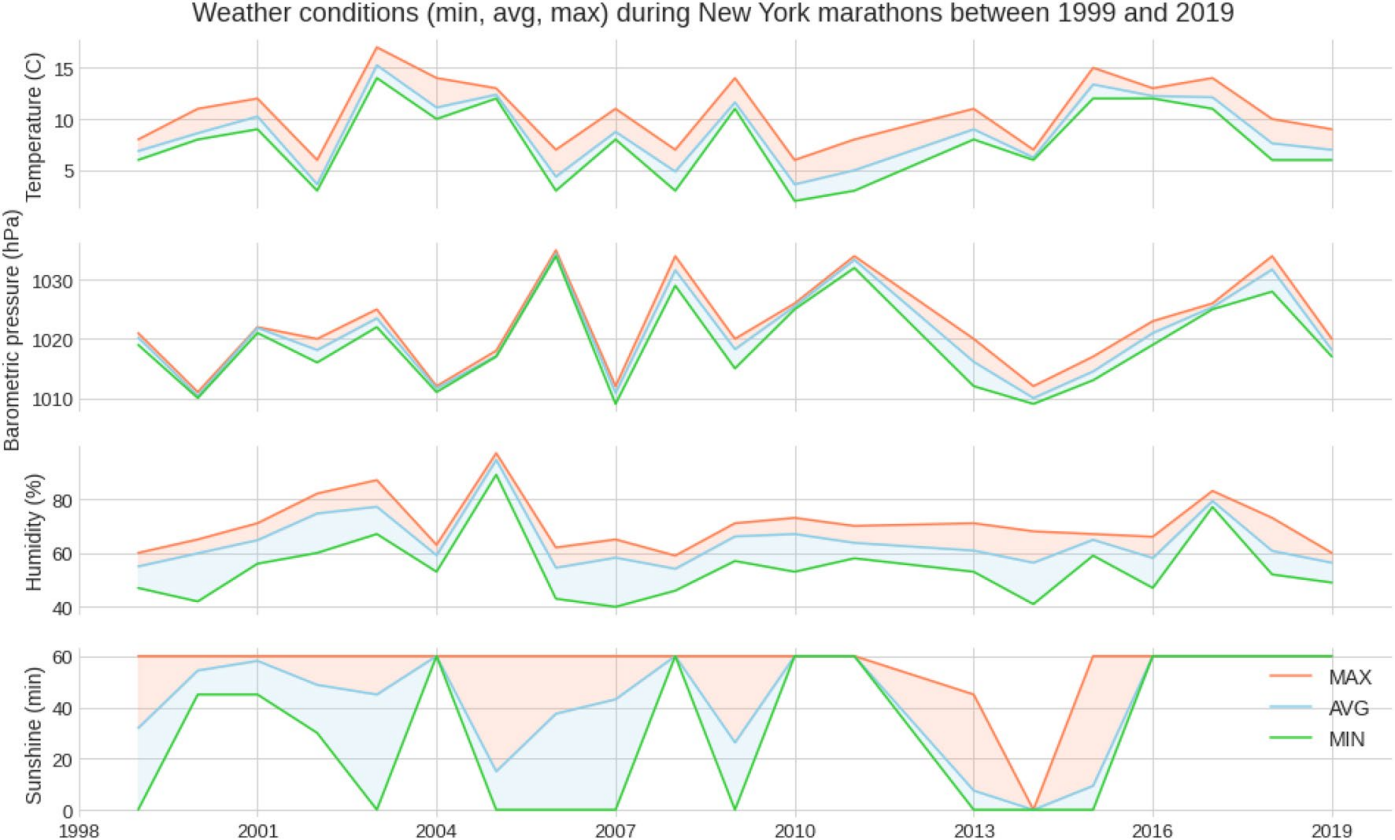
Weather conditions in the ‘New York City Marathon’ between 1999 and 2019.

**Figure 2:**
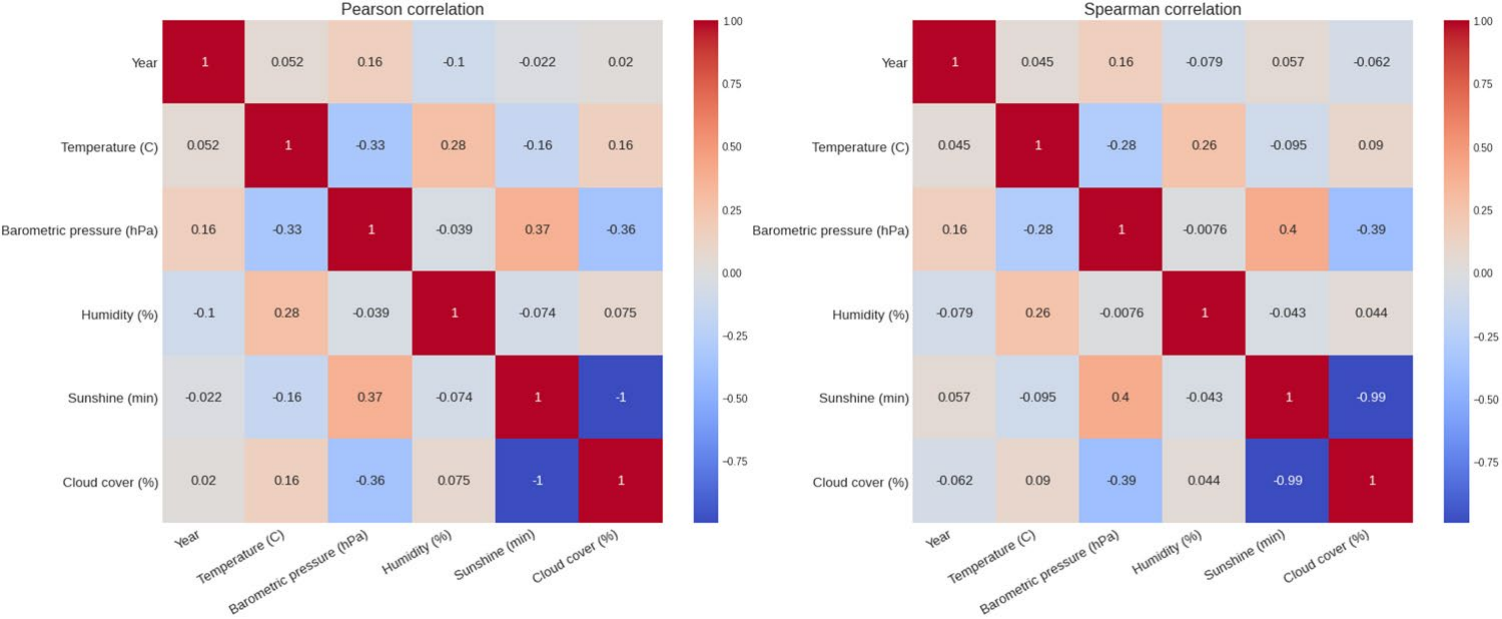
Correlation matrixes for weather variables and calendar years.

**Figure 3:**
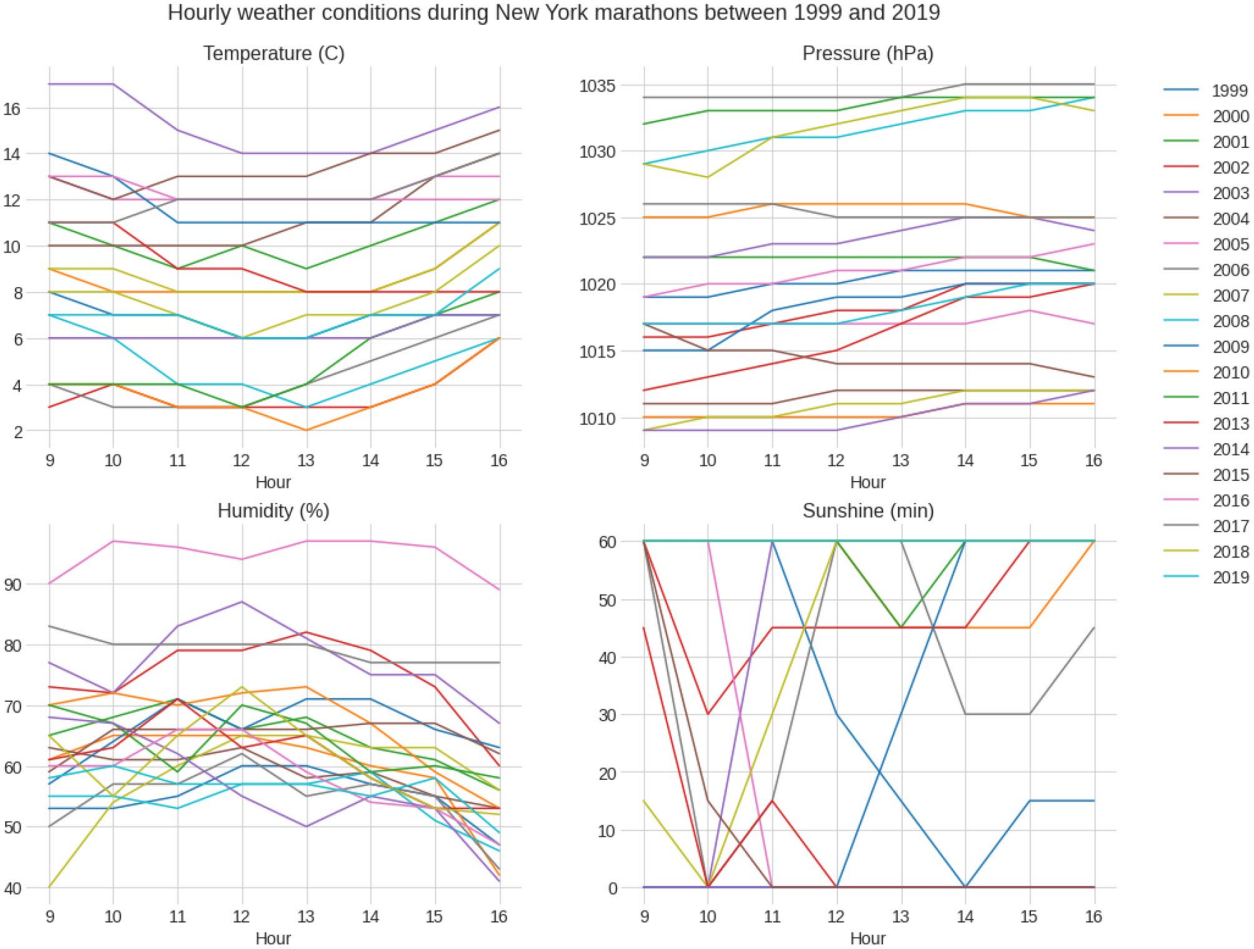
Hourly weather conditions during the ‘New York City Marathon’ between 1999 and 2019.

Figure 1 presents the weather data over the years with average, minimum and maximum values. The temperature was 8.70 (± 3.57) °C, barometric pressure 1020.69 (± 7.66) hPa, humidity 64.27 (± 11.36) % and sunshine duration during 1 race hour 42.84 (± 25.17) min with no rain during all these editions (Table 1). Figure 2 shows the correlation matrixes for weather variables and calendar years. There was a positive correlation between barometric pressure and calendar year (r = 0.16, small effect), meaning the barometric pressure has increased slightly with time. However, no similar conclusion can be drawn about temperature or humidity. In general, the temperature increased slightly over the race duration. At the same time, humidity decreased, whereas barometric pressure remained mostly unchanged (Fig. 3). This can also be observed in Fig. 1. While there is a noticeable difference between the maximum and minimum values of temperature and humidity, all three lines remain close together for the barometric pressure.

**Table 1:** Descriptive statistical for weather data.

	Temperature (°C)	Barometric pressure (hPa)	Humidity (%)	Sunshine (min)
Mean	8.70	1020.69	64.27	42.84
Std	3.57	7.66	11.36	25.17
Min	2	1009	40	0
25%	6	1014.75	57	15
50%	8	1020	63	60
75%	12	1025	70	60
Max	17	1035	97	60

Minutes of sunshine are minutes of a full hour.

**Figure 4:**
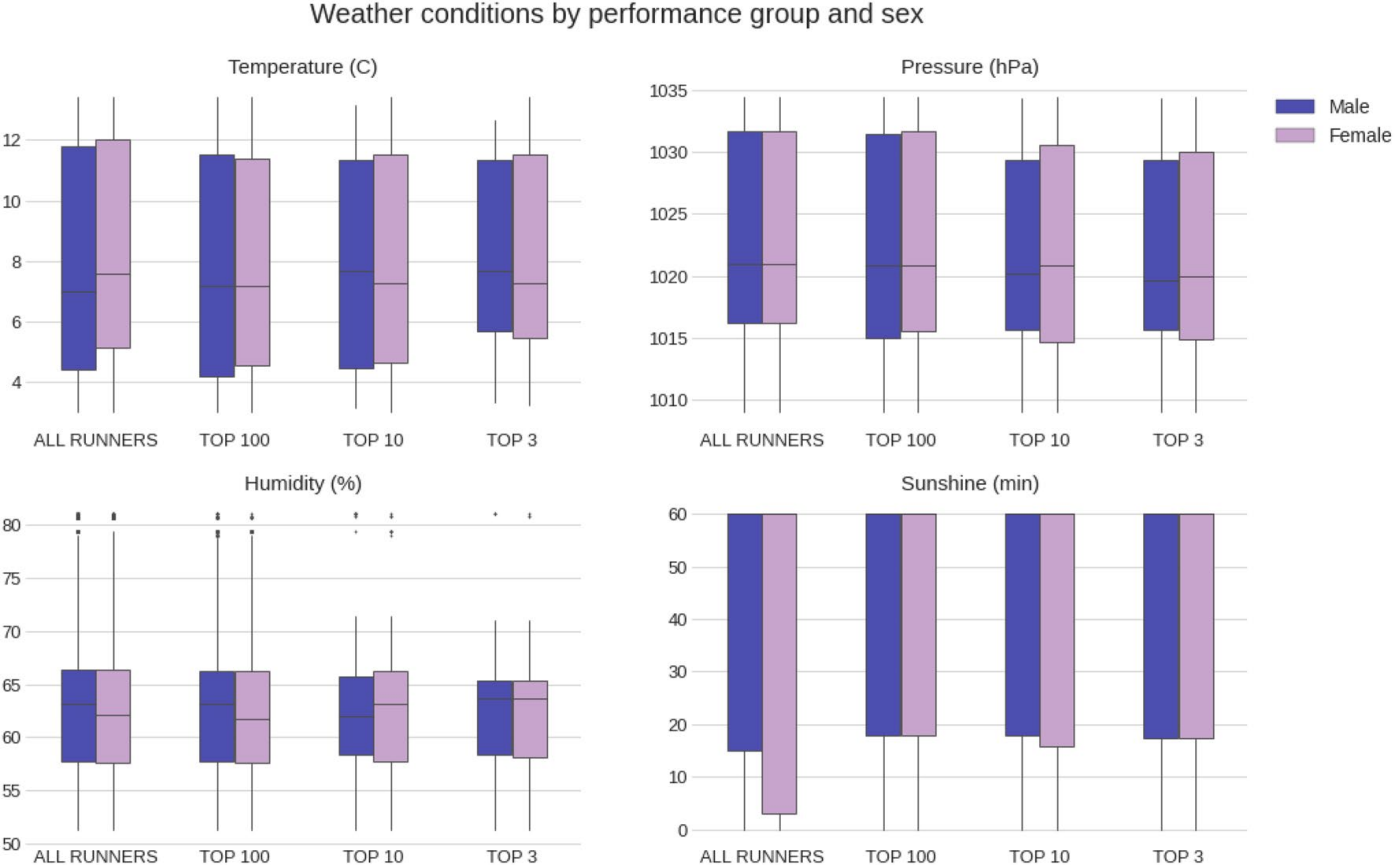
Weather conditions by performance group and sex.

Figure 4 represents the distributions of the weather conditions experienced by the different performance groups through the race for each gender separately. For each individual runner, the value of the weather factor imputed is the time-adjusted average value throughout the duration of that specific race. The values of the weather variables were not statistically significantly different between the four performance groups (p > 0.05).

### Running speed

**Figure 5:**
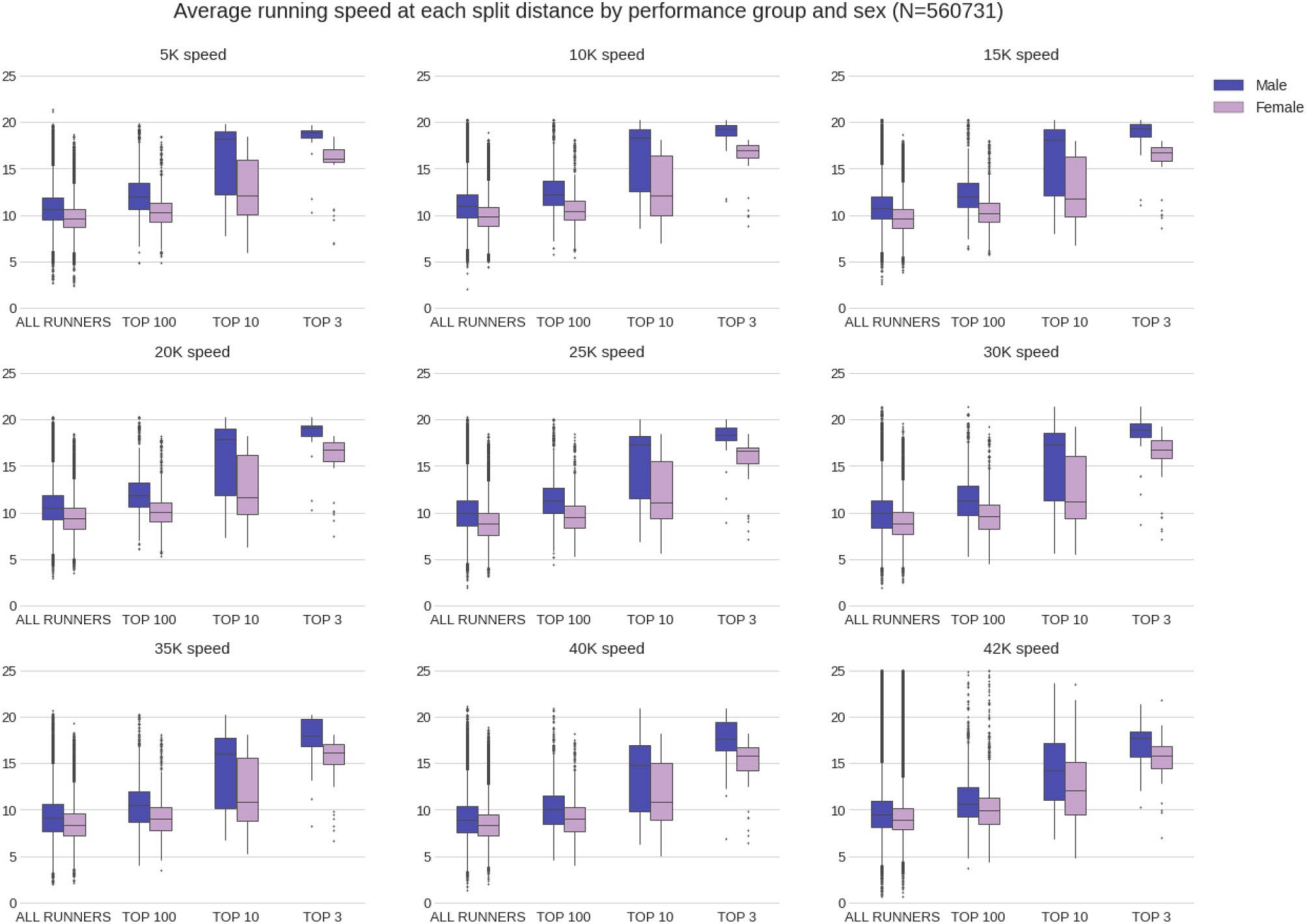
Average running speed at each split distance by performance group and sex.

Figure 5 shows the running speed for all runners, the top 100, the top 10 and the top 3, for each 5 km split. Differences in running speed between the four performance groups (all runners, top 100, top 10 and top 3) were statistically significant (p < 0.05) for both men and women.

### Association between running speed and weather

**Figure 6:**
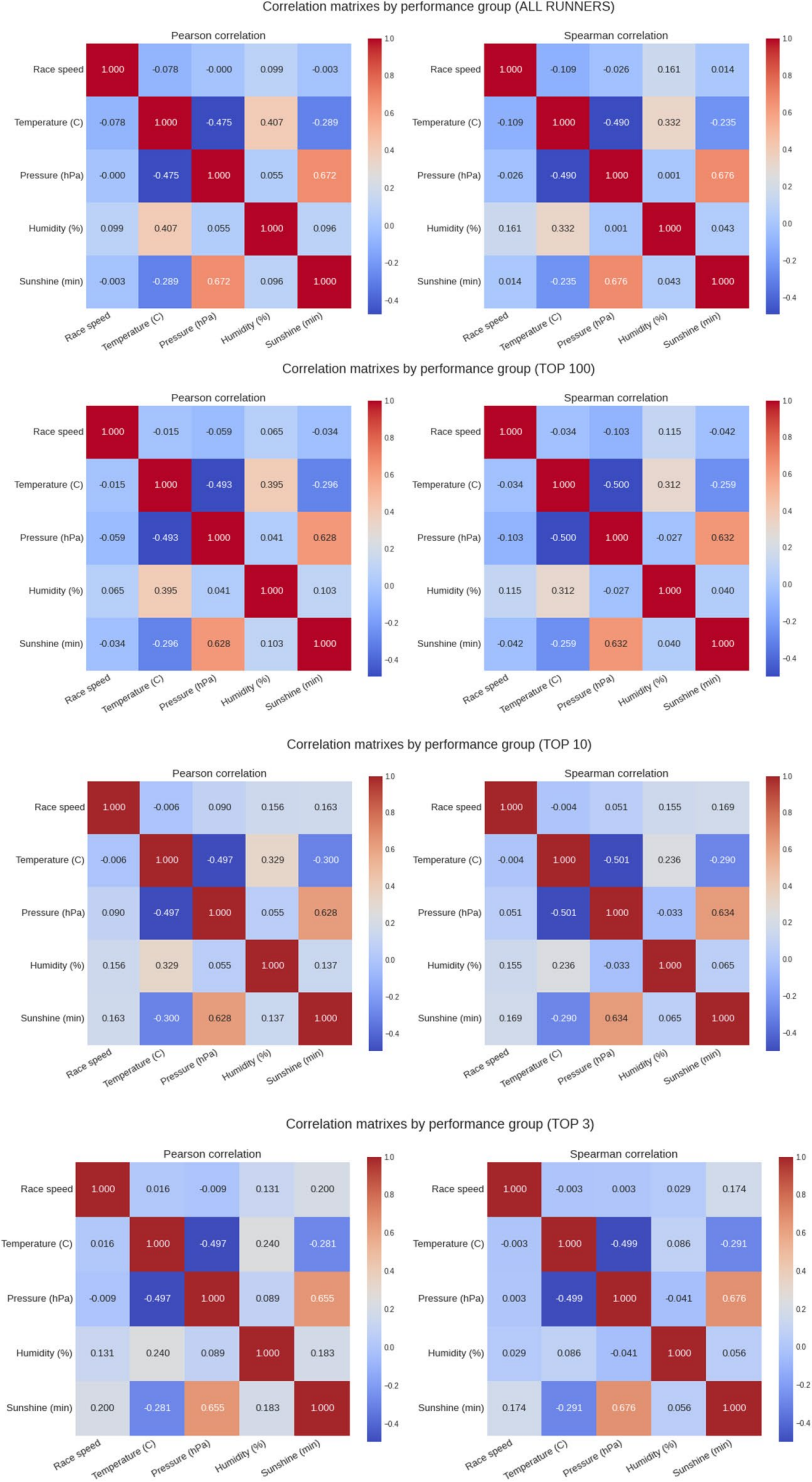
Correlation matrixes (running speed versus weather conditions) by performance groups.

**Figure 7:**
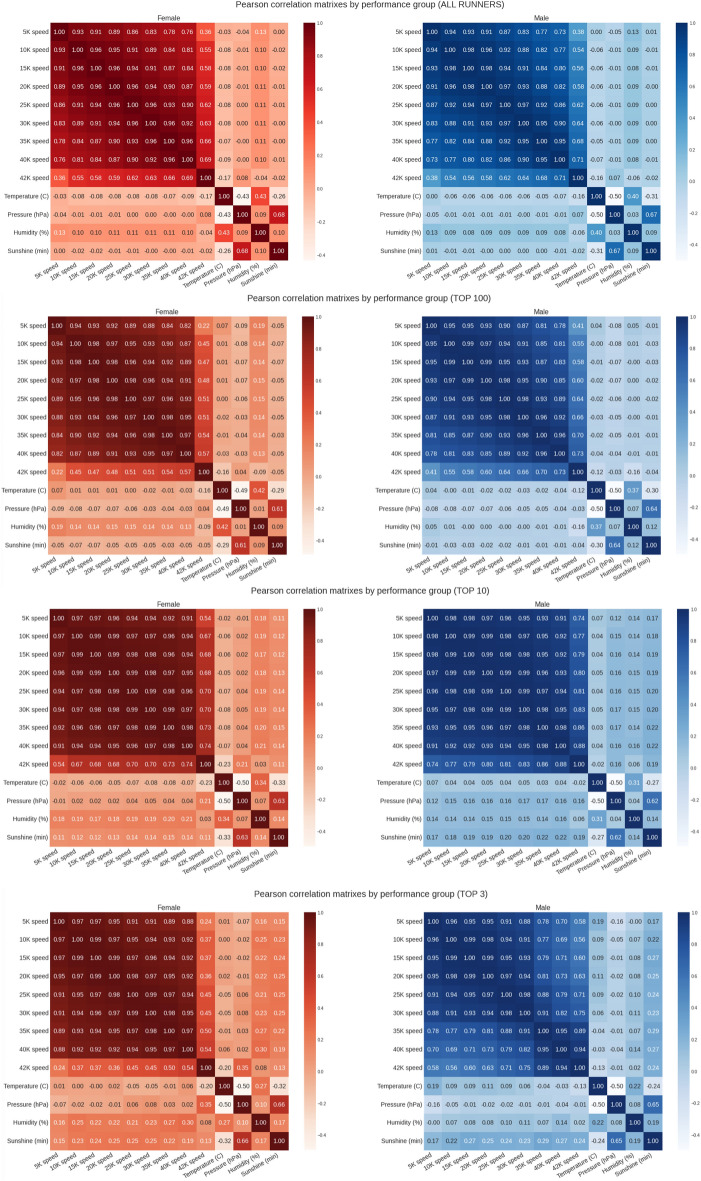
Pearson correlation matrixes by performance group.

**Figure 8:**
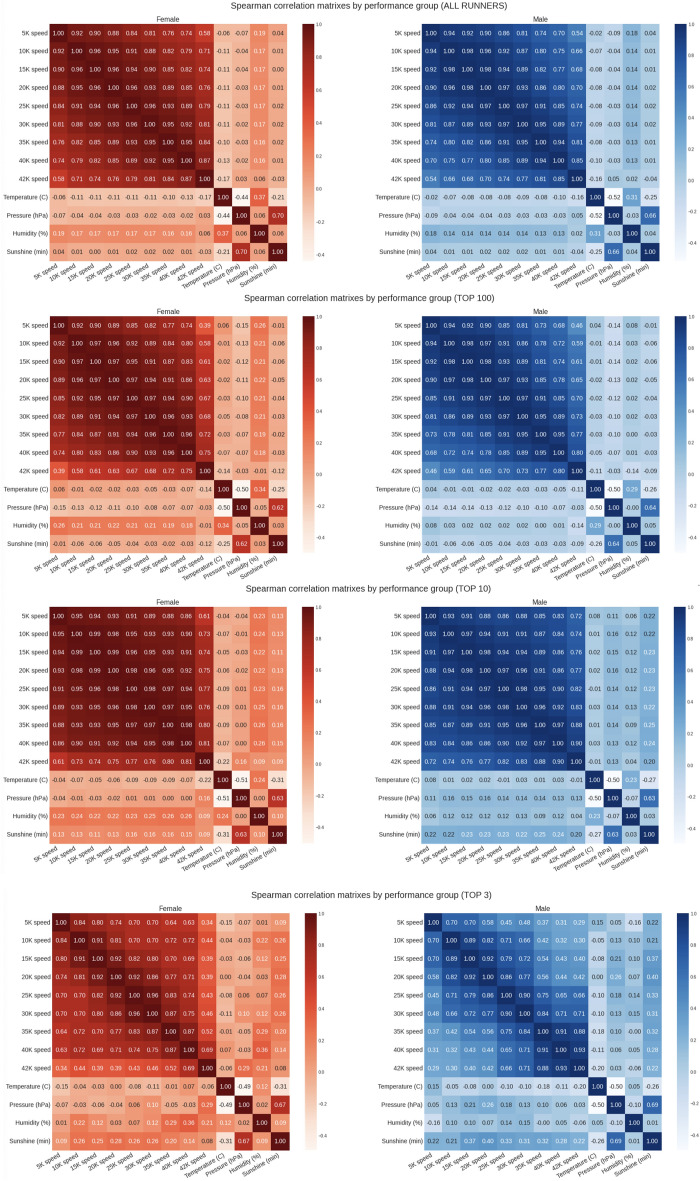
Spearman correlation matrixes by performance group.

Figure 6 presents the correlation analyses (running speed versus weather conditions) for all four performance groups. Pearson (linear) correlation was negligible for temperature and barometric pressure for all performance groups but indicated a weak and positive association with humidity in the top 10 (r = 0.15, small effect) and top 3 (r = 0.13, small effect) performance groups. This indicated that the running speed of the elite runners was positively correlated with humidity. This was similar for minutes of sunshine, where we can see a weak and positive correlation with the running speed of the elite groups [r = 0.16 (small effect) in the top 10 and r = 0.2 (small effect) in the top 3]. Spearman correlation (non-linear) identified a weak and negative correlation with temperature in all runners groups, meaning that slower running speeds were associated with higher temperatures for the entire sample. Also, non-linear positive correlation coefficients with humidity and sunshine can be observed in the Spearman matrixes, suggesting faster running speed with higher humidity and sunshine levels. Figure 7 and 8 summarize the mega matrixes by performance group, sex and correlation method to show all correlations.

### Multivariate ordinary least squares (OLS) regression analysis

A multivariate ordinary least squares (OLS) regression analysis of the average marathon speed at each split distance was performed as a function of the weather factors. Data modeling considering weather factors and race speeds yielded an R-squared of 0.035, showing no predictive power (Table 2).

**Table 2:** Multivariate ordinary least squares (OLS) regression analysis of marathon average speed at each split distance as a function of the weather factors.

OLS regression results						
Dep. variable	Y	R-squared	0.035			
Model	OLS	Adj. R-squared	0.035			
Method	Least squares	F-statistic	5101			
Date	Sat, 01 Oct 2022	Prob (F-statistic)	0.00			
Time	18:38:05	Log-likelihood	-1.1319e + 06			
No. observations	560,731	AIC	2.264e + 06			
Df residuals	560,726	BIC	2.264e + 06			
Df model	4					
Covariance type	Nonrobust					

	Coef	Std err	t	P >|t|	[0.025	0.975]
Const	31.1370	0.470	66.200	0	30.215	32.059
Temperature (°C)	−0.1131	0.001	−120.649	0	−0.115	−0.111
Pressure (hPa)	−0.0234	0.000	−50.246	0	−0.024	−0.022
Humidity (%)	0.0534	0.000	125.803	0	0.053	0.054
Sunshine (min)	−0.0011	0.000	−8.262	0	−0.001	−0.001
Omnibus	33,194.095		Durbin–Watson	0.809		
Prob (omnibus)	0.000		Jarque–Bera (JB)	41,714.737		
Skew	0.581		Prob (JB)	0.00		
Kurtosis	3.660		Cond. No	1.98e + 05 s		

## Discussion

This study intended to investigate the potential relationship between running speed and weather variables in elite runners competing in the ‘New York City Marathon’ with the hypothesis that higher temperatures negatively influence running speed in faster runners. The main findings were (i) running speed increased with increasing humidity and sunshine duration for the top 10 and the top 3 runners, (ii) running speed decreased with increasing temperature for all runners and, (iii) a positive and small correlation with humidity and sunshine duration where running speed decreased with higher levels of humidity and sunshine for all runners.

### Elite runners became faster with increasing humidity and sunshine duration

We could not confirm our hypothesis that the fastest runners would slow down with increasing temperature since the running speed increased with increasing humidity and sunshine duration for the top 3 and top 10 runners. A study investigating marathoners of different performance levels competing in the ‘Berlin Marathon’ between 1974 and 2019 showed that elite runners (i.e., top 3 and top 10 runners) became faster with increasing temperatures^26^.

The most likely explanation here is that the temperature variation within the first 2 or 3 h of each event is very small. When we check Fig. 3, we see that most temperature changes occur from 13:00 h onwards. Elite runners starting the race at 09:00 and completing it within 2 or 3 h experienced little temperature change (insufficient to manifest any effect on their performance).

An interesting aspect for these elite runners was that they became faster with increasing sunshine duration. The aspect of sunshine duration has also been investigated in the ‘Berlin Marathon’ for world record performances. For male world record performances, the ideal environmental conditions were temperatures of 18.61 °C, sunny, mostly dry days, with higher atmospheric pressure and little cloud cover^27^^.^ The most likely explanation is that the fastest runners in large city marathons such as the ‘New York City Marathon’ originate from East Africa (Kenya, Ethiopia) and are used to training and running in warmer environments^2^.

### Overall runners became slower with increasing temperature

A second important finding was that running speed decreased for overall runners with increasing temperature. A decrease in running speed due to increasing temperatures is a common finding^28^. It has been shown that increasing temperatures can slow down faster and slower runners^22,29^. For example, in three Japanese women’s championship marathons, increasing air temperatures slowed running speed more in faster runners (i.e., winners, 25th) than slower runners (50th, 100th)^30^. In age group marathoners competing in the ‘New York City Marathon’ between 1970 and 2019, performance was lower on days with higher temperatures where higher temperatures affected more older runners (40–59 years old men and 25–65 years old women)^21^. In contrast, elite runners (i.e., annual top 3 and annual top 10 runners) competing in the ‘Berlin Marathon’ between 1974 and 2019 became faster with increasing temperatures^26^. The fastest marathon runners in the ‘Berlin Marathon’ achieved their fastest race times on race days with higher maximum temperatures of 15–30 °C^31^. The disparate findings for slower and faster runners might be explained by methodological differences, such as different sample sizes, race courses and environmental conditions. We must, however, also be aware that a decrease in marathon running speed is also due to progressive damage in the skeletal muscle^32^.

### Overall runners became slower with increasing humidity and sunshine duration

A third important finding was that running speed decreased with increasing humidity and sunshine duration. This is in contrast with the top 3 and the top 10 runners. The influence of humidity on marathon running performance has been investigated^21,28,33,34^; however, only a few studies found an influence on marathon running performance^21^. A study investigating data from 1,280,557 finishers in the ‘New York City Marathon’ between 1970 and 2019 showed that humidity was negatively associated with race times, where men were significantly more affected than women. The effect of high humidity on marathon running performance was increased in 40–59 years old men and 25–65 years old women^21^. Overall, humidity does not significantly influence marathon running performance, whereas temperature has a higher impact^34^. More studies are needed to investigate the influence between running performance and humidity.

The limitations of the present study include the lack of control for morphological (e.g., body mass and body fat) and physiological indexes, training (e.g., training planning, deliberate practice), marathon experience, and hydration during the race. Another important limitation is the missing Wetbulb Global Temperature (WBGT) which is not provided in the historical weather data. For this, we suggest caution in the generalization of the results. However, we have analyzed data covering different performance levels in one of the most important race events around the world, which can offer insights for athletes training for the New York City Marathon.

## Conclusions

In elite runners competing in the ‘New York City Marathon’ between 1999 and 2019, running speed slightly increased with the increased humidity and sunshine duration for the top 10 and the top 3 runners, while overall runners became slower with increasing temperature, humidity and sunshine duration. Weather factors affected running speed and results but did not provide significant predictive influence on performance, highlighting that training, body composition and nutrition must be considered better predictors of runners' performance. Key takeaways for athletes and coaches are that running speed increased with increasing humidity and sunshine duration for elite runners, but decreased with increasing temperature, higher humidity and sunshine levels for all runners.

## Data Availability

The datasets used and analysed during the current study are available from the corresponding author on reasonable request.
